# Computer-aided indirect bonding versus traditional direct bonding of orthodontic brackets: bonding accuracy and bonding failures. A split mouth randomized controlled trial

**DOI:** 10.1093/ejo/cjag067

**Published:** 2026-09-18

**Authors:** Izabela Czolgosz, Paolo M Cattaneo, Malgorzata Sowinska, Marie A Cornelis

**Affiliations:** Ludwik Rydygier Collegium Medicum in Bydgoszcz. Department of Maxillofacial Orthopaedics and Orthodontics, Nicolaus Copernicus University, Jagiellońska 13-15 Street, 85-067 Bydgoszcz, Poland; Melbourne Dental School, Faculty of Medicine Dentistry and Health Sciences, The University of Melbourne, 720 Swanston Street, Carlton, VIC 3053, Australia; Private Practice, Polna 7B Street, 87-100 Torun, Poland; Melbourne Dental School, Faculty of Medicine Dentistry and Health Sciences, The University of Melbourne, 720 Swanston Street, Carlton, VIC 3053, Australia

**Keywords:** Orthodontics, indirect bonding, computer-aided design, randomized controlled trial

## Abstract

**Objectives:**

The aims of this randomized controlled trial were to compare the accuracy of computer-aided indirect bonding with conventional direct bonding, and to evaluate bracket survival.

**Methods:**

Consecutive patients were randomly allocated to two groups (blocks of four, online generated sequence) using a split-mouth design with a computer-aided indirect and a direct bonding method: group 1 (upper right and lower left quadrants: computer-aided indirect bonding; upper left and lower right quadrants: direct bonding) or group 2 (opposite situation). Modified ABO-OGS scores were calculated at the end of treatment. Bracket bonding failures, as well as bracket rebondings and finishing bends because of inaccurate bracket positioning were recorded at every visit. The primary outcome was bracket placement accuracy. The secondary outcome was bracket survival. Outcome assessment was blinded. To compare bracket placement accuracy, paired t-tests were used together with linear mixed models. For the survival analysis, a piecewise Cox model was used.

**Results:**

Thirty-seven patients were randomized to group 1 or group 2. Twenty-seven patients were analyzed for bracket survival, and 26 for ABO-OGS scores. There was no significant difference (*P* = 0.76, 95% CI: −1.18, 0.87) in mean modified ABO-OGS scores between both methods. Among the brackets bonded with computer-aided indirect bonding, 66 brackets (27%) needed rebonding or finishing bends because of inaccurate positioning. Fifty-six brackets bonded with direct bonding (23%) were rebonded or required finishing bends because of inaccurate bracket positioning. The bonding method did not appear to significantly influence the proportion of teeth needing rebonding/wire bending because of inaccurate bracket positioning. A total of 44 out of 532 brackets got debonded throughout treatment: 22 debonded brackets were totalized with each bonding method (8.3%). There was no statistically significant difference in survival between both bonding techniques. The hazard ratio (HR) computer-aided indirect bonding to direct bonding during the first year was estimated at 1.15 (*P* = 0.67, 95% CI: 0.61, 2.17). After the first year, the HR indirect to direct was estimated at 0.46 (*P* = 0.38, 95% CI: 0.09, 2.53). No harm was observed.

**Conclusions:**

There were no statistically significant differences in bonding accuracy and in bracket survival between computer-aided indirect and direct bonding methods.

**Registration:**

This trial was retrospectively registered on ClinicalTrials.gov (University of Aarhus Protocol Record 10101).

**Protocol:**

The protocol was not published before trial commencement.

## Introduction

Accurate bracket positioning is the key to effective orthodontic treatment [1]. The ideal position and angulation of teeth is defined according to Andrew's six keys to perfect occlusion [2]. The straight wire concept is based on proper bracket placement, and preadjusted brackets are mostly used today by clinicians [3, 4]. Incorrect bracket placement affects the final tooth position: it can affect tooth alignment in all planes of space and can lead to increased chair time for rebonding or archwire adjustments, extended treatment times, and inadequate results [5]. Studies comparing the accuracy of bracket placement between direct and indirect bonding methods have found some inaccuracies with both bracket bonding methods [4, 6, 7]. Evaluation of accuracy of bracket placement *in vivo*, both for direct and indirect bonding methods, is limited and difficult [4]. Aguirre *et al*. and Koo *et al*. investigated bracket placement precision with indirect and direct bonding methods. They found improvement in vertical position with indirect bonding, although there was no difference in angulation and mesiodistal position of brackets [4, 6]. On the contrary, other authors did not find any difference in bracket position accuracy between both techniques [7–9].

Using high-tech procedures involving a computer-aided process where the operator can place virtual brackets on a virtual 3D model, could possibly reduce positioning errors and increase the precision of this ‘digital’ indirect bonding method [10]. Computer-aided technology is commonly used today in different fields of dentistry and application of this method is promising. Computer-aided bracket placement on virtual models and 3D printed trays for transferring brackets to the mouth of the patients seems very attractive, the technology is already used in many practices and will continue to increase with improvement of the method. However, using transfer trays for indirect bonding might also generate limitations and difficulties for orthodontists, such as the impossibility to correct the bracket position during bonding or removing the composite excess around the bracket because of the presence of the tray during the bonding procedure [11]. Moreover, extra laboratory steps and increased cost still limit the use of the technique in daily practice [12, 13]. Computer-aided transfer trays have been found to be acceptable *in vitro*, and clinical trials support this finding [14–16]. Chaudhary compared CAD/CAM and PVS transfer trays for indirect bonding: CAD/CAM trays exhibited greater accuracy, except in the vertical dimension [17]. Schwarzler *et al*. evaluated the transfer accuracy and immediate loss rate of rigid (hard) versus flexible (soft) 3D printed indirect bonding trays and found that soft resin is more favorable than hard resin for accuracy and usability [16].

With regards to bracket survival rate, most of the studies showed no significant differences when comparing indirect and direct bonding methods. The total bracket failure rate was found to range from 1.4% to 10.7% [6, 18–23]. However, a more recent systematic review compared failure rate between both techniques and concluded that direct bracket bonding technique has a lower failure rate than the indirect technique in the long term (12–15 months) [24].

Two systematic reviews have reviewed studies investigating 3-printed trays and concluded that CAD/CAM printed trays have an acceptable accuracy rate but concluded that there is no strong evidence and that more clinical studies are needed [14, 25].

Therefore, the evaluation of bonding accuracy and failures of bracket bonding with computer-aided indirect bonding is relevant for future orthodontic daily practice.

### Specific objectives and hypotheses

The objectives were to assess and compare the accuracy of an indirect computer-aided bonding method with a conventional direct bonding method, and to evaluate bracket survival related to both bonding methods.

The null hypotheses were that there is no difference in accuracy of bracket placement between the direct and indirect computer-aided bonding methods, and that bracket bond failure is similar for both bonding methods.

## Participants and methods

### Trial design and any changes after trial commencement

This single-center, 2-arm, parallel, randomized controlled trial with a 1:1 allocation ratio used a split-mouth design. No changes occurred after trial initiation.

### Participants, eligibility criteria, and settings

The sample and settings were previously described [13]. Consecutive patients were enrolled from 30 April 2015 until 30 September 2017 at the Section of Orthodontics, Department of Dentistry and Oral Health, provided that they required full fixed appliance according to treatment needs and accepted to have metallic brackets. The inclusion criteria comprised: the presence of minimum four permanent teeth (except molars) to be bonded in each quadrant and all teeth fully erupted. The exclusion criteria were: patients with teeth presenting active caries, fluorosis or hypoplasia of enamel, restorations or fractures of the surfaces to be bonded, or abnormalities in crown morphology, as well as patients with major rotations impeding proper bracket positioning. Syndromic conditions and skeletal discrepancies were also excluded. The patients were informed about the study verbally and in written. Patients (or a parent or guardian in case of a minor patient) were asked to sign an informed consent before bonding. This study was approved by the Ethical Committee of Research of the Human Being of Central Jutland, Denmark (reference number: 1-10-72-120-15).

### Interventions

Digital bracket placement and clinical bonding procedures (direct and indirect) were performed by 10 calibrated orthodontic postgraduate residents, with the help of a trained dental assistant. The patients were randomly allocated to group 1 (upper right and lower left quadrants bonded with indirect bonding, and upper left and lower right quadrants with direct bonding); or group 2 (upper left and lower right quadrants bonded with indirect bonding, and upper right and lower left quadrants with direct bonding). If extractions were part of the treatment plan, they were postponed after bonding. The direct and indirect bonding methods were previously described in details [13]. All patients had metallic brackets (LowProfile System, American Orthodontics, Sheboygn, Wisconsin, USA) bonded on incisors, canines, and premolars. A sealed envelope was delivered to the operator with the information of the split-mouth set-up according to the randomization, before digital bracket placement. Digital bracket placement was done with the DDP-Ortho software (version 1.6_2917, Czestochowa, Poland) and bonding trays were 3D printed (IPA, Czestochowa, Poland) with a rigid-elastic plastic material (Visijet StonePlast, MP200 Plastic Material, 3D Systems Europe, UK). An OPG was available during the bonding sessions with both bonding techniques, as well as during digital bracket placement for the indirect bonding technique. The bite was raised with bite blocks in case of presence of premature contacts on the brackets in maximal intercuspation.

#### Accuracy of bracket placement

Two assessors (I.C., M.S.) calculated American Board of Orthodontics objective grading system (ABO-OGS) scores for the two quadrants bonded with indirect bonding (from last premolar to last premolar, excluding the molars), as well as for the two quadrants bonded with direct bonding (from last premolar to last premolar, excluding the molars), based on end-of-treatment models produced by intraoral scanning (Trios 3, 3Shape, Copenhagen, Denmark) and subsequently printed; as well as on a panoramic radiograph taken at the end of treatment [26]. The ABO-OGS calculation was then further modified to exclude the three ABO-OGS components involving occlusion, namely overjet, occlusal contacts, and occlusal relationships. The scores of the 2 quadrants bonded with the direct method were summed up to give a score for the direct method, while the scores of the 2 quadrants bonded with the indirect method were summed up to give a score for the indirect method. The two assessors had previously been trained and calibrated using the original ABO calibration kit. The measurements were obtained using the ABO gauge.

Moreover, the number of brackets rebonded because of inaccurate positioning, as well as the number of finishing bends needed because of unsatisfactory bracket positioning were recorded at every orthodontic visit, until the end of treatment.

#### Bracket survival

Bracket bonding failures were recorded at each appointment until debonding, as well as at any intermediate emergency appointment throughout the treatment: type of tooth, as well as type of interface failure (bracket-composite or composite-enamel) was recorded. Only first time failures were taken into account. The failed brackets were replaced by new brackets bonded with the direct technique.

### Outcomes (primary and secondary) and any changes after trial commencement

The primary outcome of the present investigation was accuracy of bracket placement with the CAD/CAM indirect and the traditional direct bonding techniques. The secondary outcome was the survival of brackets. No changes were made after trial commencement.

### Sample size calculation

The previously described sample size was calculated according to the primary outcome of the initial publication of this randomized controlled clinical trial: bonding time [13].

### Randomization

The randomization method was previously described [13]. Briefly, randomization in blocks of four was applied using a sequence generated online (randomization.com), to group 1 or group 2, as described above, with different split-mouth set-ups. Allocation concealment was ensured by the use of sealed envelopes.

### Blinding

Blinding of either patient or operator was not possible during bracket bonding. However, accuracy outcome assessment was blinded: anonymization was ensured by assigning a number to each patient.

### Statistical analysis

To compare the bonding accuracy, the modified ABO scores for both techniques were compared with a paired-sample *t*-test. Linear mixed models with two crossed random effects of subjects and raters were fitted for the ABO scores, adjusted for two confounders: age and sex of patient. The fitting method was restricted maximum likelihood with a Kenward-Roger approximation [27]. For comparing the number of brackets rebonded because of inaccurate positioning, and the number of finishing bends needed because of unsatisfactory bracket positioning, the teeth having brackets rebonded because of bond failures were excluded. A Generalized Linear Mixed Model was fitted to model a binary outcome with a multilevel clustered data structure. Correlation was induced between teeth in the same mouth by fitting a subject-specific model using the default Gaussian-Hermite quadrature method with 30 integration points [28]. Fixed effects evaluated were age, sex, bonding method, tooth number, whereas patient was fitted as a random effect. After this, predicted marginal probabilities for bonding method and tooth number were obtained by integrating out over the range of the random effect of patient [29]. For the survival analysis, a piecewise Cox model was fitted, in which non proportional hazards were modeled by assuming proportional hazards in a series of consecutive time intervals (the database was split into two consecutive intervals: from 0.5 to 365 days and from day 366 onward) [30], which was achieved using the technique of time varying covariates. This piecewise Cox model met the proportional hazards assumption in either time intervals.

### Error of the method

The ABO-OGS scores were assessed twice by each operator at a 2-week interval in order to determine intra- and inter-observer reliability. Linear mixed models were fitted to hierarchical data with two crossed random effects of subjects and raters. Separate models were fitted for each bonding method, adjusting for the confounders age and sex of patients. The fitting method was restricted maximum likelihood with a Kenward-Roger approximation [27]. All correlation coefficients and limits of agreement were then computed using estimates of variance components from the above models.

## Results

### Participant flow

**Figure 1:**
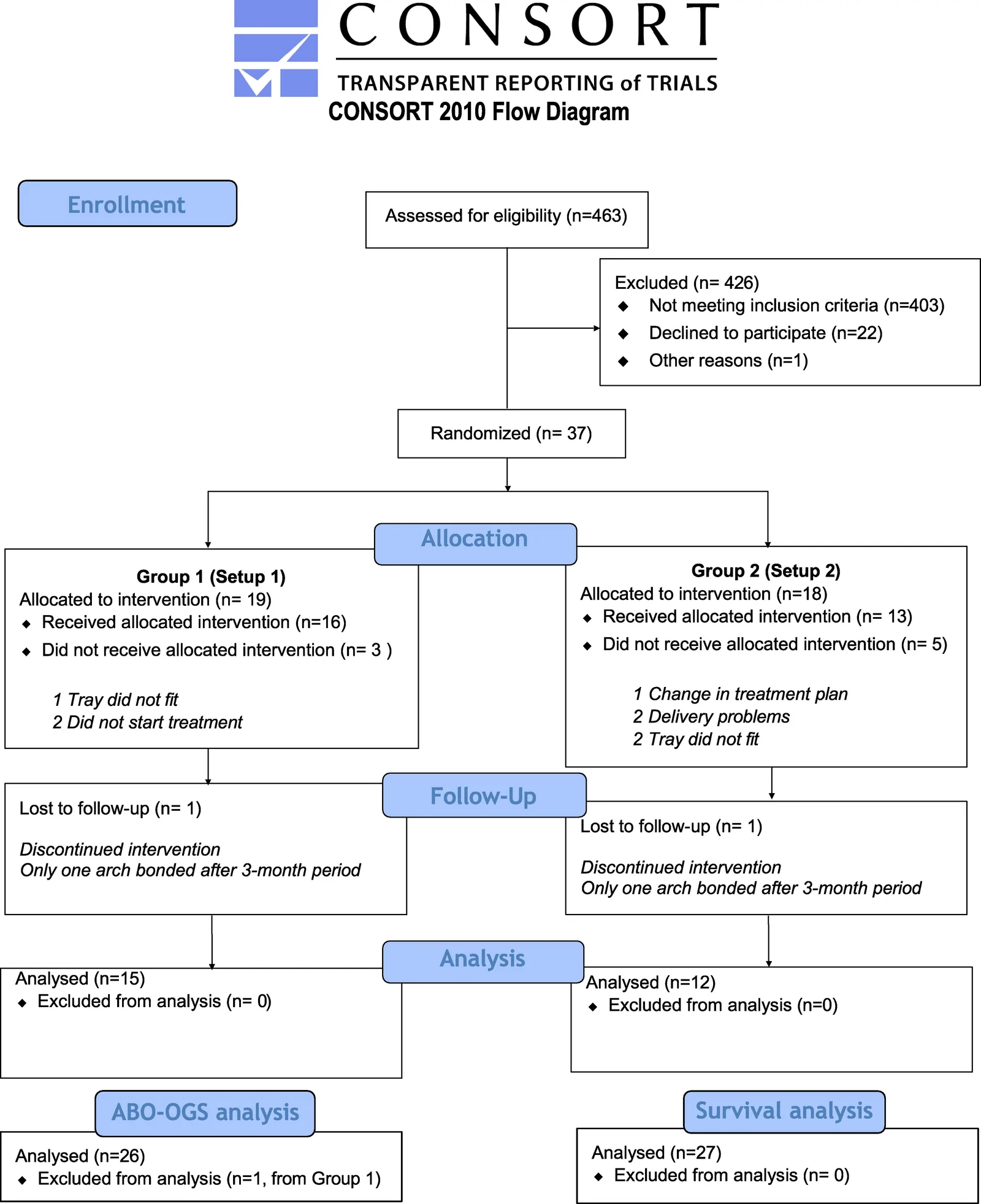
CONSORT diagram showing the flow of patients through the trial.

As previously described [13], 37 patients were randomized to either group 1 or group 2 (CONSORT flow diagram, Fig. 1) Eight patients did not receive the allocated intervention, and two patients were lost to follow-up [13]. Twenty-seven patients, 15 in group 1 and 12 in group 2, were analyzed for bracket survival. Twenty-six patients were included in the ABO-OBS analysis, as the panoramic radiograph had not been taken for one patient.

### Baseline data

The baseline characteristics of the patients (age, sex and type of extraction—non extraction treatment) were very similar in both groups, as reported previously [13].

### Primary outcome: accuracy of bracket placement

#### ABO-OGS

There was no significant difference [*P* = 0.76, 95% confidence interval (CI): −1.18, 0.87] in mean modified ABO-OGS scores between computer-aided indirect bonding and traditional direct bonding [mean scores, for two quadrants: computer-aided indirect bonding 6.15, standard deviation (SD) 2.02; direct bonding 6.00, SD 2.42]. Confounders age and sex were both statistically significant. The mean ABO-OGS scores for two quadrants increased by 0.14 units for every 1 year of age (*P* = 0.02, 95% CI: 0.03, 0.26). The mean ABO-OGS scores were significantly higher (*P* = 0.025, 95% CI: 0.34, 4.62) for males than for females (predicted mean scores, for two quadrants: females 4.52 (95% CI: 1.72, 7.32); males 7.00 (95% CI: 4.1, 9.9)).

**Figure 2:**
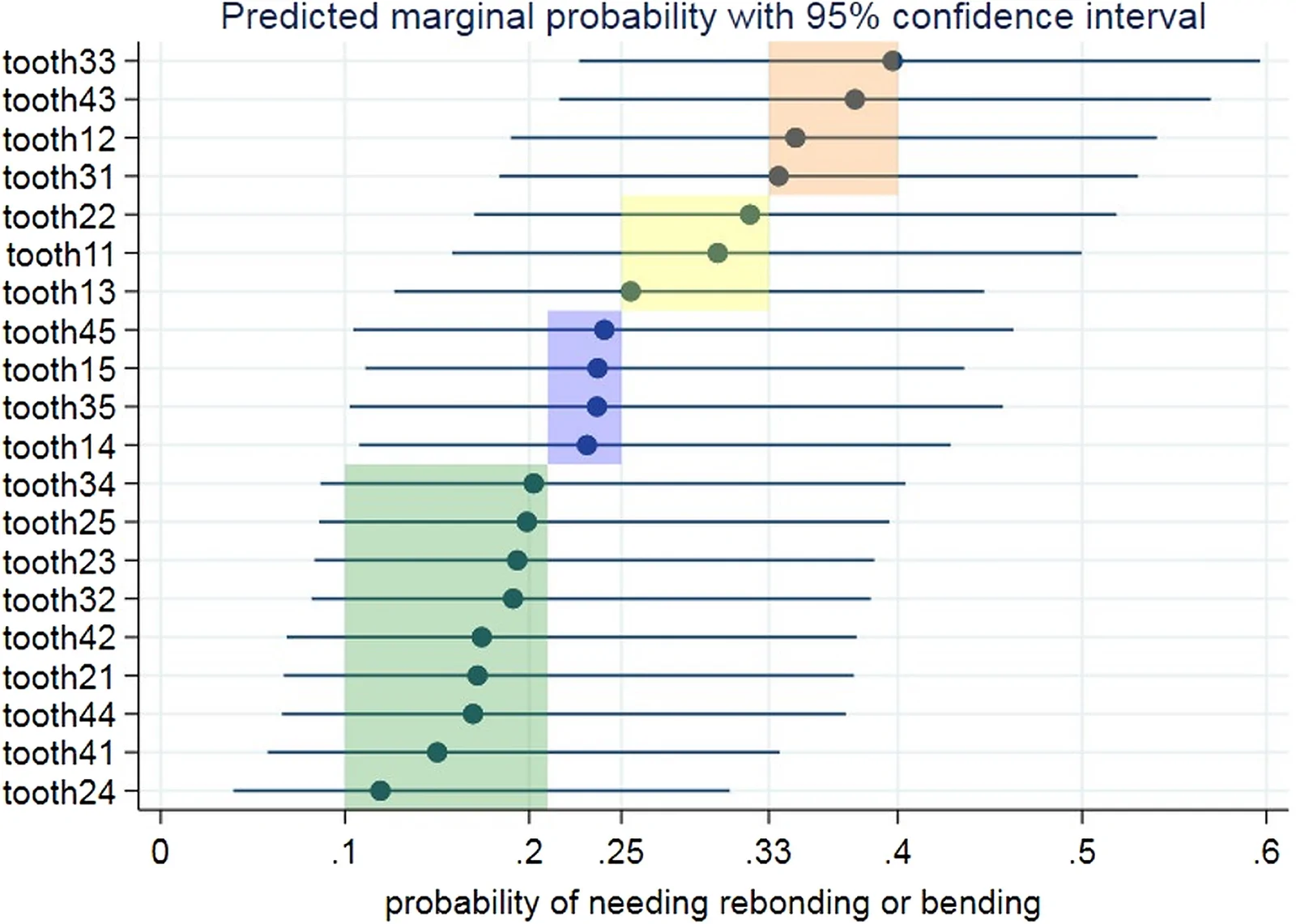
Predicted marginal probability of teeth needing rebonding/wire bending because of inaccurate bracket positioning, with 95% confidence intervals. The teeth in the green shaded area have a predicted probability of needing rebonding/wire bending no higher than one in five, the teeth in the blue shaded no higher than one in four, the teeth in the yellow shaded area no higher than one in three, and the teeth in the orange shaded area no higher than two in five.

In total, 532 brackets were bonded. Forty-four brackets were excluded because they were rebonded due to bond failures, leaving 488 analyzed brackets: 244 were bonded with each bonding method. Fifty-six brackets bonded with direct bonding (23%) were rebonded or required finishing bends because of inaccurate bracket positioning. Among the brackets bonded with computer-aided indirect bonding, 66 brackets (27%) needed rebonding or finishing bends because of inaccurate positioning. Adjusted for the effect of age and of gender, the bonding method did not appear to significantly influence the proportion of teeth needing rebonding/wire bending because of inaccurate bracket positioning, and neither did tooth number. Teeth bonded with direct bonding were predicted to need rebonding/wire bending with a probability of 0.23 (95% CI: 0.17, 0.31). For teeth bonded with computer-aided indirect bonding, the corresponding predicted probability was 0.25 (95% CI: 0.19, 0.33) [Odds ratio direct/indirect bonding: 0.89 (95% CI: 0.60, 1.31)]. The teeth predicted to need rebonding/wire bending because of inaccurate bracket positioning with a chance higher than at least one in three were teeth 33, 43, 12, and 31 (Fig. 2).

### Secondary outcome: bracket survival

**Figure 3:**
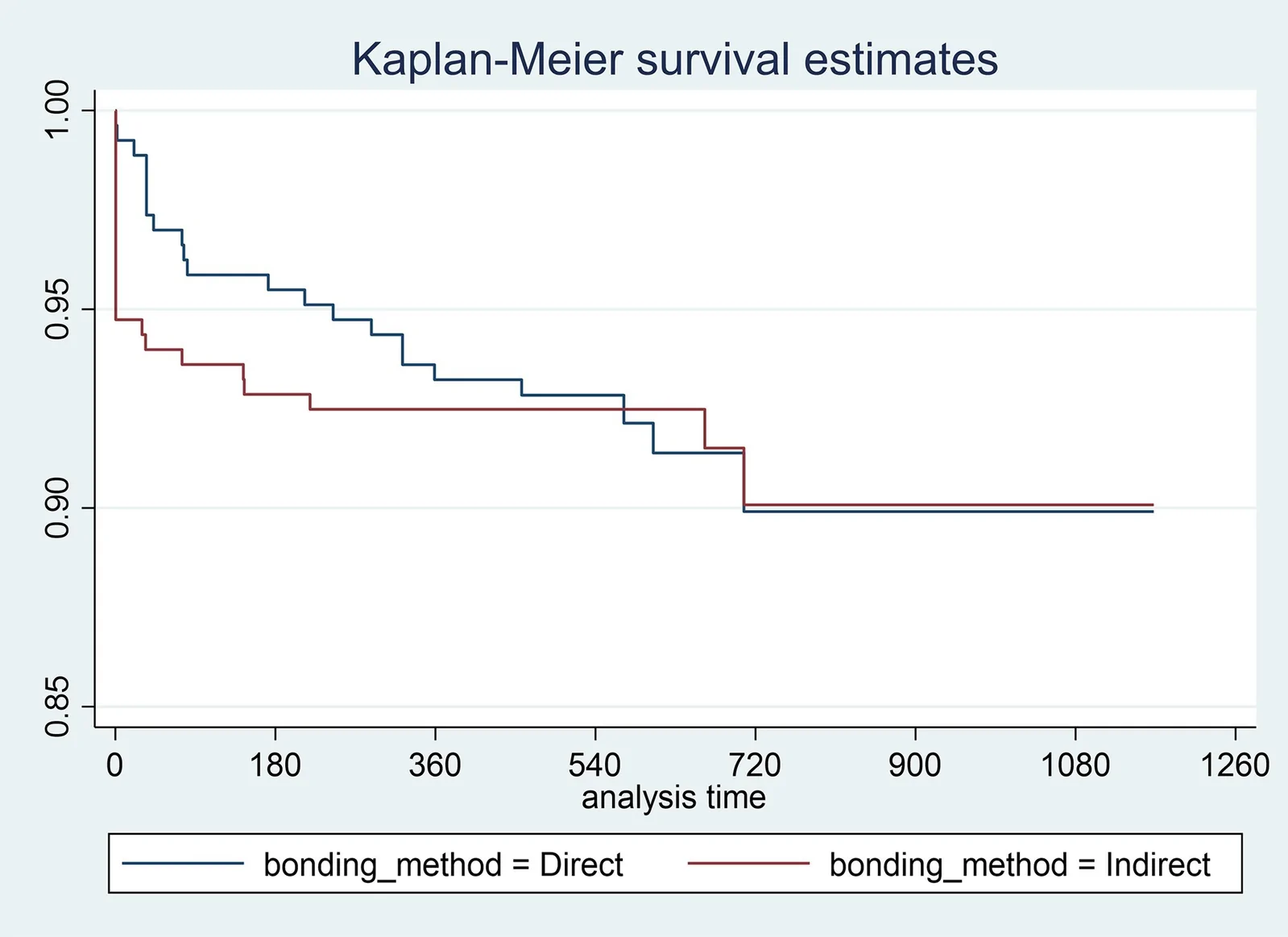
Kaplan-Meier survival estimates.

A total of 44 out of 532 brackets got debonded throughout treatment, including the immediate debondings reported previously [13]: 22 debonded brackets were totalized with each bonding method (22/266: 8.3%). There were 15 debondings in the upper arch (5 with direct and 10 with indirect bonding) and 29 in the lower arch (17 with direct and 12 with indirect bonding). Twenty-three brackets failed at the enamel-composite interface (12 with direct bonding and 11 with indirect bonding), and 21 at the bracket-composite interface (10 with direct bonding and 11 with indirect bonding). The Kaplan-Meier survival curves are shown in Fig. 3. There was no statistically significant difference between both bonding techniques. Confounders age and sex were not statistically significant. Computer-aided indirect bonding method had a slightly higher risk of debonding during the first year of treatment compared with direct bonding, while it was the opposite after the first year: The hazard ratio (HR) indirect bonding to direct bonding during the first year was estimated at 1.15 (*P* = 0.67, 95% CI: 0.61, 2.17). After the first year, the HR indirect to direct was estimated at 0.46 (*P* = 0.38, 95% CI: 0.09, 2.53).

### Error of the method

**Figure 4:**
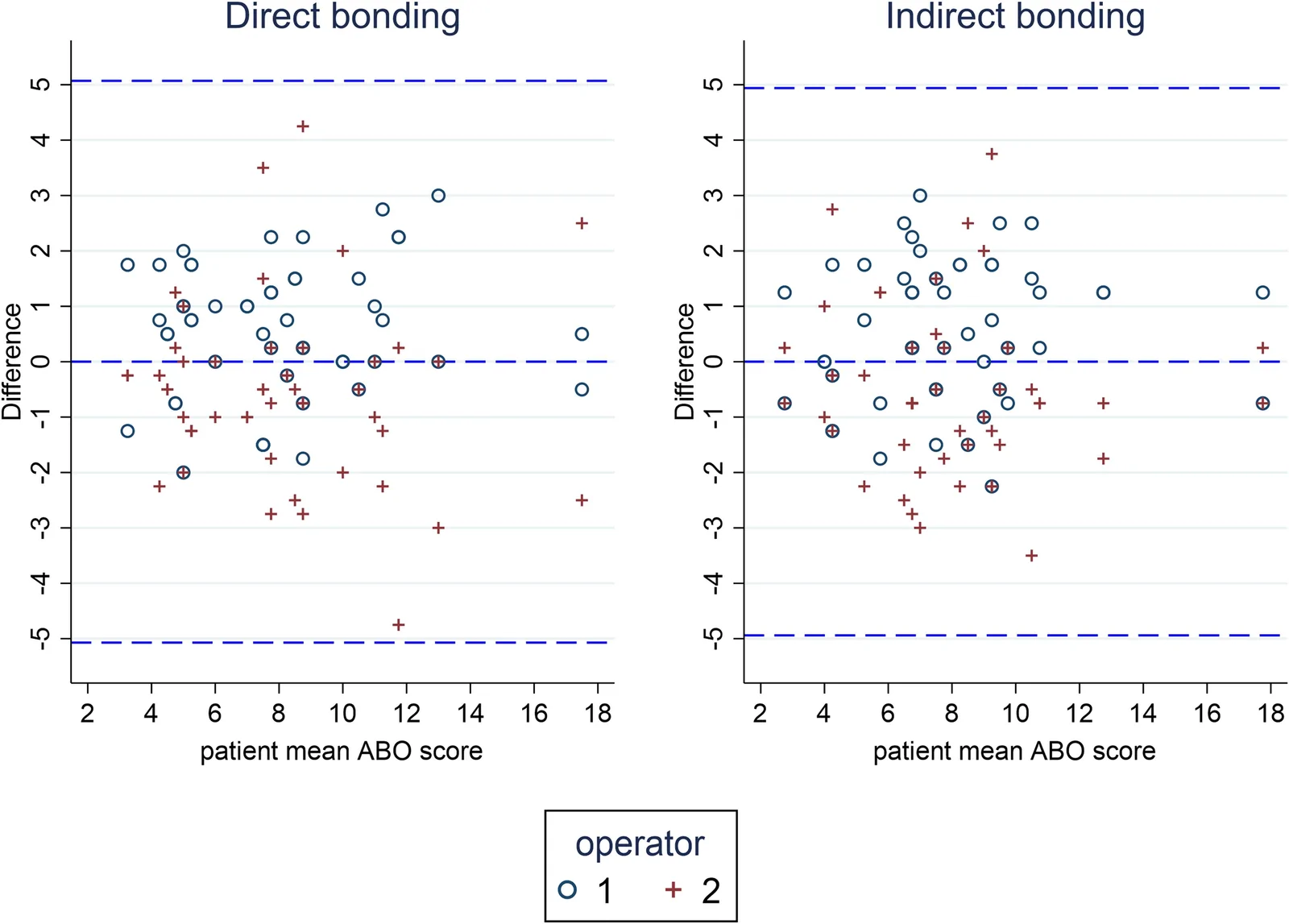
Limits of agreement.

Components of variance of total ABO-OGS scores (including all components) for the direct bonding method were estimated as: subject 7.83 (95% CI: 4.18, 14.64), operator systematic error 0.64 (95% CI: 0.03, 12.65), measurement error 2.57 (95% CI: 1.87, 3.52). Components of variance for the computer-aided indirect bonding method were estimated as: subject 5.84 (95% CI: 3.08, 11.08), operator systematic error 0.60 (95% CI: 0.03, 11.88), measurement error 2.46 (95% CI: 1.80, 3.37). Limits of agreement were ±5.07 for direct bonding and ±4.94 for indirect bonding. No differences were found outside the limits of agreement (Fig. 4).

### Harms

No harm was observed.

## Discussion

### Main findings in context of existing evidence

This randomized controlled trial is the first one comparing bonding accuracy and bracket bonding survival of CAD/CAM indirect bonding and traditional direct bonding of orthodontic brackets. The study showed that there was no significant difference in the overall accuracy of bracket placement between the CAD/CAM indirect bonding and the direct bonding methods, based on ABO-OGS, the numbers of finishing bends and brackets rebonded because of positioning errors. These findings are in agreement with clinical trials published by Zachrisson *et al*. and Hodge *et al*. based on a traditional indirect bonding where accuracy was evaluated using a photographic method [7, 31]. The authors concluded that the number of bracket placement errors were similar compared with direct bonding [7, 31]. On the other hand, Yildrim and co-workers demonstrated that leveling of marginal ridges were significantly better with traditional indirect bonding than with direct bonding [32]. Better vertical positioning with traditional indirect bonding was also found by Koo *et al*. [4].

Recent studies show that CAD/CAM indirect trays can transfer bracket position from the digital set-up with high accuracy and clinical acceptable results [14, 33–37]. Chaudhary and co-workers found 3D printed trays being more accurate than PVS trays used for traditional indirect bonding, except in the vertical dimension. The vertical errors in bracket positions with 3D printed trays might be due to errors in 3D model orientation in the software, or to the rigidity of the resin used for fabrication of the 3D trays compared with PVS material [17, 38]. The orientation of the 3D models in the printer used for printing indirect trays could also be considered to influence the precision, which was tested by Supple and co-workers. They found no statistically significant difference in the accuracy of indirect 3D printed trays based on models oriented with 15 or 75 degrees angulation [39]. Furthermore, the design of the tray can play a role in the accuracy of bracket transfer from the virtual model to the mouth of the patient. In the present study, the tray had no full coverage, the jigs were partially covering the brackets, and the insertion of the tray into the mouth was quite easy. Glasenapp *et al*. tested two 3D printed tray designs and their influence on the transfer accuracy [40]. Their trays mainly differed in retention mechanism for the brackets and their overlap (jig shape vs pocket shape), and they found that both designs were clinically suitable and provided acceptable bracket placement accuracy [40]. Moreover, Karabiber and Eglenen tested the transfer accuracy of bar versus shell design CAD/CAM trays in a randomized clinical trial and concluded that there was no statistical difference between both trays [37]. Similar findings were reported by Ueno *et al*. in another randomized clinical trial from 2025 [34]: They found that CAD/CAM indirect bonding for self-ligating and conventional brackets showed similar treatment success [34].

In the present study, 10 postgraduate residents performed all the bondings. Clinical experience could influence the accurate positioning of the orthodontic brackets, thus lead to a better final result. De Oliveira *et al*. demonstrated that clinical experience only had a positive influence on accuracy in the angular dimension for virtual bonding [41]. Interestingly, they also found that postgraduate students were more precise in positioning brackets vertically compared with experienced clinicians [41]. Armstrong *et al*. also showed that accurate direct bonding of orthodontic brackets does not appear to be related to clinical experience [42].

In the present study, ABO-OGS scores were higher for males than females. ABO-OGS scores were also higher in young patients compared with adult patients, with scores increased by 0.14 units for every one year of age. Patient cooperation and motivation is a significant factor for achieving good final results during orthodontic treatment. Barbosa *et al*. reported that a greater cooperation was expected from adult individuals who presented lower bracket failure rate [43]. Larsson and co-workers demonstrated that 52% of adolescents do not follow the treatment instructions provided by the orthodontist, which could explain why in the present study younger patients finished with higher ABO-OGS scores than adults [44].

The teeth predicted to need rebonding/wire bending because of inaccurate bracket positioning with a chance higher than at least one in three were teeth 33, 43, 12, and 31. The lower canines are very often rotated and overcorrection could be needed to get a proper tooth position, likewise incisors are often worn or crowded and bracket placement might be more challenging in those teeth.

The present study demonstrated that bracket failure rates were similar between both bonding methods. Bonding failures rates of 8.3% are in agreement with previous studies and are clinically acceptable according to Vijayakumar *et al*. [23, 45]. The present results are also similar to findings of Read and O’Brien, who investigated the failure rate of indirect bonding using light-cured adhesives and reported an overall bracket failure rate of 6.5% [18].

Schwärzler and co-workers compared the immediate failure ratę of hard versus soft CAD/CAM indirect bonding trays in a clinical trial [16]. The failure rate was 2.4% in the hard resin tray group and 2.3% in the soft resin tray group [16].

The present investigation showed that most of the bracket failures occurred in the lower jaw, which is in agreement with Bozelli *et al*., and might be attributed to occlusal forces [21]. During indirect bonding, it is sometimes difficult to place the transfer tray with even pressure to all teeth, especially when a vacuum-formed transfer tray is used, so the type of tray used might have an influence on bracket survival [36, 45, 46].

In the present study, computer-aided indirect bonding method had a slightly higher risk of debonding during the first year of treatment compared with direct bonding, while it was the opposite after the first year. This finding can be related to the high number of immediate debondings, and to the learning curve of the CAD/CAM method [13]. No failure was observed with the indirect bonding method between ∼6 months and 2 years.

### Limitations

The limitations related to the split-mouth design and the number of participants of this randomized trial was previously discussed [13].

This trial was a single-center trial. One limitation of this study certainly includes the limited experience of the postgraduate students, with individual learning curves and skills. Indirect bonding technique seems to be very sensitive to errors at every stage and learning curve could affect the number of debondings. That said, the operators were trained and calibrated on patients prior to the commencement of the trial.

Sample size calculation was performed based on bonding time [13]. Potentially the sample size might not have been appropriate to detect differences in accuracy of bracket placement or bracket survival.

Attrition was rather high, which may increase the risk of bias.

The modification of the ABO-OGS score excluding the molars and the 3 components involving interarch relations can also be seen as a potential limitation.

Although blinding of the operator was not feasible at the intervention stage, outcome assessment was blinded; therefore, the risks of observation and detection biases can be considered low.

To overcome these limitations, multicenter trials with experienced clinicians and larger sample sizes would be recommended in the future.

### Generalizability

Due to the fact that the brackets were bonded by postgraduate residents, the results of this study might not reflect the situation of an experienced clinician using the CAD/CAM indirect bonding method to treat patients on a daily basis.

## Conclusion

The result of this randomized controlled trial shows that:

- There is no difference in bonding accuracy between CAD/CAM indirect bonding and traditional direct bonding.
- There is no significant difference in bracket failure rates between computer-aided indirect bonding and traditional direct bonding.

## Data Availability

Data available on request.
